# Explainable Dynamic Graph Learning and Multi-Scale Feature Fusion for Hydraulic System Health Monitoring

**DOI:** 10.3390/s26051478

**Published:** 2026-02-26

**Authors:** Ziheng Gu, Xiansong He, Yibo Song, Gongning Li, Shufeng Zhang, Xiaowei Yang, Xiaoli Zhao, Jianyong Yao, Chuanjie Lu

**Affiliations:** 1 School of Mechanical Engineering, Nanjing University of Science and Technology, Nanjing 210094, Chinayaojianyong@njust.edu.cn (J.Y.); 2 Key Laboratory of Intelligent Operation and Maintenance of High-Parameter Lifts, State Administration for Market Regulation, Special Equipment Safety Supervision Inspection Institute of Jiangsu Province, Suzhou 215000, China; 3 National Key Laboratory of Equipment State Sensing and Smart Support, College of Intelligence Science and Technology, National University of Defense Technology, Changsha 410073, China; 4 Dezhou Precion Machine Tool Co., Ltd., Shandong Precion Group, Dezhou 253799, China

**Keywords:** hydraulic system, Dynamic Multi-Scale Graph Neural Network, fault diagnosis, multi-scale feature extraction, dynamic graph learner

## Abstract

Hydraulic systems are pivotal components in safety-critical aerospace and industrial applications, making reliable health monitoring essential. However, traditional data-driven diagnosis methods typically rely on static graph structures that fail to capture evolving sensor correlations during different fault modes. Furthermore, existing grid-based models often struggle to extract multi-resolution features and maintain performance under data-limited conditions. To address these challenges, this paper proposes a novel Dynamic Multi-Scale Graph Neural Network (DMS-GNN) for hydraulic system fault diagnosis. The framework integrates a hierarchical multi-scale feature extraction module to capture diverse fault signatures across different frequency bands. Crucially, a self-attention-based dynamic graph learner is introduced to adaptively infer latent sensor topologies end-to-end, eliminating the reliance on predefined physical connections. Experimental validation on a dedicated electro-hydraulic test bench demonstrates that the proposed DMS-GNN achieves a superior diagnostic accuracy of 98.47%, outperforming state-of-the-art baselines such as GraphSAGE, Static GCN, and GAT. The result confirms the efficacy of combining multi-scale temporal learning with dynamic spatial reasoning for robust multi-sensor fusion diagnosis.

## 1. Introduction

Hydraulic systems have emerged as the indispensable actuation solution in modern energy applications [1], aerospace flight control systems [2,3], and high-end manufacturing equipment [4]. With their superior power-to-weight ratios, rapid dynamic responses, and ability to handle heavy loads, hydraulic systems are irreplaceable in scenarios requiring high-force actuation [5,6]. However, the complex coupling between mechanical, hydraulic, and electrical subsystems makes them susceptible to various faults, such as internal leakage, sensor drift, and nonlinear parameter uncertainties [7,8]. These failures, if not detected in their incipient stages, can lead to irreversible performance degradation or catastrophic safety accidents [9]. Therefore, developing accurate, robust, and interpretable fault diagnosis techniques is an imperative requirement for ensuring the reliability of safety-critical equipment.

In the past decades, fault diagnosis methodologies have been extensively studied, generally categorized into model-based and data-driven approaches. Model-based methods rely on establishing precise mathematical models (e.g., state estimation, parameter identification) to monitor residuals between observed and predicted states [10,11]. For hydraulic systems, researchers have developed various analytical redundancy and advanced control techniques. For instance, Yang et al. proposed neural adaptive dynamic surface control [12] and appointed-time performance control [13] to guarantee transient performance and system stability. Others employed Active Disturbance Rejection Control (ADRC) [14] and adaptive sliding mode observers [15] to detect anomalies. Although effective for simple linear systems, these methods face significant challenges in practical applications due to strong nonlinearities (e.g., friction, flow saturation) and parameter uncertainties under varying working conditions [16]. Consequently, constructing a high-fidelity physical model that covers all operating modes is often impractical.

To bypass the reliance on physical parameters, data-driven approaches, particularly Deep Learning (DL), have gained increasing attention [17,18]. Powered by the capability to learn hierarchical representations from massive monitoring data, algorithms such as Convolutional Neural Networks (CNNs) [19,20], Transformer architectures [21], and Deep Attention Convolutional Capsule Networks [22] have become the de facto standards for processing industrial time-series data. For instance, Xing et al. [23] proposed a hybrid data-driven fusion network for electro-hydraulic fault diagnosis, and Hu et al. [24] developed an intelligent decoupling diagnosis method for compound faults in EHAs. Generative models and diffusion-based zero-shot learning approaches have also been explored to handle small sample scenarios [25,26]. While these methods have achieved remarkable success, they typically treat multi-sensor data as simple multi-channel images or independent sequences, inherently ignoring the non-Euclidean spatial dependencies among heterogeneous sensors.

In practical hydraulic monitoring scenarios, multiple heterogeneous sensors—such as pressure transducers, displacement sensors, and flow meters—are deployed to capture the system state from different physical domains [27]. The correlations among these sensors contain rich diagnostic information. However, effectively fusing these heterogeneous data streams remains a formidable challenge due to two fundamental limitations in existing works. The first limitation lies in single-scale feature extraction. Fault signatures in hydraulic systems manifest across different frequency bands; for example, mechanical impacts typically cause high-frequency vibrations, while internal leakage often results in low-frequency pressure trends. Standard fixed-kernel convolutions struggle to capture these multi-scale patterns simultaneously [28,29]. The second limitation involves the static assumption of sensor topology. Most existing methods assume that the relationship between sensors is static or independent. In reality, the topological dependency among sensors is dynamic and evolves with the fault mode. For instance, the correlation between motor current and outlet pressure might be strong during a jamming fault but weak during a sensor bias fault. Traditional graph methods based on physical connections or Pearson correlation [30] fail to model these evolving dependencies.

To address these challenges, this paper proposes a Dynamic Multi-Scale Graph Neural Network (DMS-GNN). Unlike conventional methods that rely on predefined graph structures, DMS-GNN introduces a dynamic graph learner module that adaptively infers the latent topology of the sensor network end-to-end. By combining this with a multi-scale temporal feature extraction mechanism, the proposed model can effectively capture both the diverse frequency characteristics of fault signals and the evolving spatial dependencies among heterogeneous sensors.

The main contributions of this work are summarized as follows:

(1) A novel Dynamic Multi-Scale Graph Neural Network (DMS-GNN) is developed for hydraulic system fault diagnosis. This framework unifies multi-scale temporal representation learning and spatial graph reasoning into an end-to-end trainable architecture, significantly enhancing feature discriminability.
(2) A self-attention-based dynamic graph learner is proposed to replace static graph construction methods. This module automatically learns the adjacency matrix from data, allowing the model to adaptively highlight critical sensor correlations specific to different fault modes without requiring expert knowledge.
(3) Comprehensive experimental validation is conducted on a dedicated electro-hydraulic experimental test bench. The results demonstrate that DMS-GNN achieves superior diagnostic accuracy (over 96%) and robustness compared to state-of-the-art CNN and static GCN baselines. Additionally, the proposed framework provides insights into predictive uncertainty and feature importance, enhancing its practical interpretability.

The remainder of this paper is organized as follows. Section 2 reviews related work. Section 3 details the proposed DMS-GNN method. Section 4 describes the experimental setup and results. Section 5 discusses the experimental findings and limitations. Finally, Section 6 concludes the paper.

## 2. Related Work

### 2.1. Deep Learning Based Fault Diagnosis

With the advent of Industry 4.0, deep learning has been widely adopted for the Prognostics and Health Management (PHM) of complex machinery [17,18]. Among various architectures, 1D-CNNs are particularly favored for their ability to extract local shift-invariant features from vibration or pressure signals. To handle the diverse frequency components of fault signals, multi-scale CNN architectures have been proposed. For instance, Jiang et al. [28] applied multi-scale CNNs to enhance feature learning for wind turbine gearboxes, demonstrating that aggregating features from different receptive fields improves classification performance. Similarly, Yin et al. [31] developed a multi-scale graph convolutional framework for rolling bearing diagnosis.

Beyond CNNs, Transformer architectures have been introduced to PHM to weigh the importance of different time steps via self-attention mechanisms [21,32]. Despite their effectiveness in temporal modeling, these grid-based models treat sensors as isolated channels or simple vectors. They fail to explicitly model the interaction logic (e.g., energy flow or causal relationships) between different components of the hydraulic system, which limits their interpretability and performance in multi-sensor fusion tasks. More recently, advanced generative architectures have emerged to address data scarcity; for example, He et al. [26] proposed a diffusion-enhanced dual-domain adversarial network for zero-shot fault diagnosis, demonstrating superior generalization in unseen fault modes.

### 2.2. Graph Neural Networks in PHM

**Figure 1:**
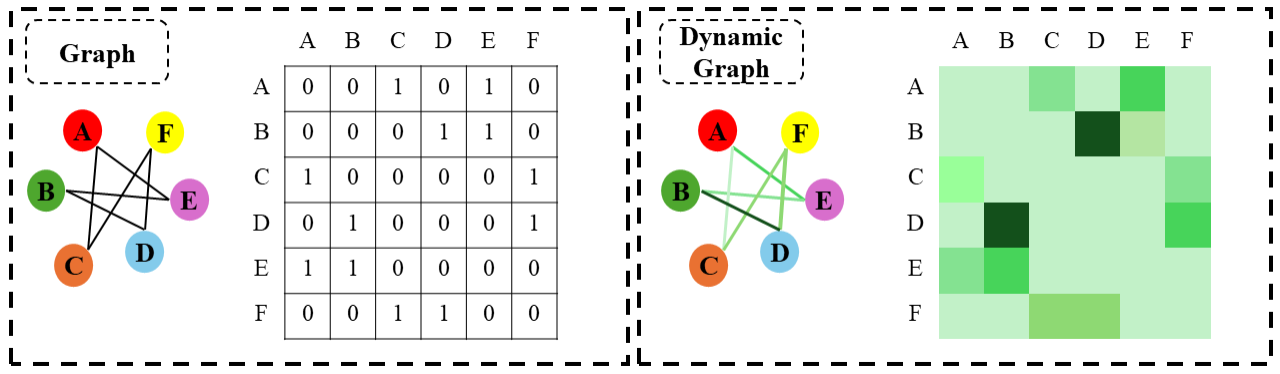
Motivation of the proposed method. The comparison illustrates the difference between the traditional static graph (**left**) and the proposed dynamic graph (**right**). Unlike static methods that use a fixed topology, our dynamic graph learner adaptively highlights critical sensor correlations specific to different fault modes.

To address the limitations of grid-based models, Graph Neural Networks (GNNs) have been introduced to process non-Euclidean data by defining sensors as nodes and their dependencies as edges [33,34]. GNNs, including Graph Convolutional Networks (GCNs) [35] and Graph Attention Networks (GATs) [36], utilize message-passing mechanisms to aggregate information from neighboring nodes. In the context of fault diagnosis, Zhao et al. [30] proposed a semi-supervised graph convolutional deep belief network, constructing a graph based on the similarity of samples. Chen et al. [37] utilized GCNs for domain adaptation. Other works define the graph structure based on the prior physical connectivity of the system. While these methods incorporate spatial information, they typically rely on predefinedand staticgraph structures. As noted in recent studies on multivariate time series [38], a fixed graph structure cannot accommodate the dynamic changes in system operating conditions. For example, in a hydraulic system, the coupling strength between hydraulic pressure and motor current varies significantly between different fault modes. A static adjacency matrix leads to suboptimal information fusion when the actual sensor correlations deviate from the predefined rules (as illustrated in Figure 1).

### 2.3. Dynamic Graph Learning

To overcome the reliance on predefined priors, dynamic graph learning has emerged as a promising direction in spatiotemporal forecasting and anomaly detection. The core idea is to learn the graph structure (i.e., the adjacency matrix) end-to-end from the data itself. Seminal works such as the Multivariate Time Series Graph Neural Network (MTGNN) [38] and Graph Deviation Network (GDN) [39] have demonstrated that learning a latent graph structure can significantly improve anomaly detection performance. Li et al. [40] further proposed dynamic graph structure learning for forecasting tasks.

Building upon these findings, recent studies have rapidly advanced dynamic spatiotemporal graph learning for machinery fault diagnosis. For instance, Yang et al. [41] developed a dynamic graph-driven method that adaptively updates relationship information to capture time-varying spatial dependencies in rotating machinery. Similarly, Zhang et al. [42] proposed a multiscale channel attention-driven graph dynamic fusion network to achieve robust fault diagnosis under noisy industrial environments. Furthermore, dynamic graph convolutional networks have been increasingly leveraged to model the temporal dependencies of multi-sensor data, proving that continuously updating the adjacency matrix can effectively adapt to shifting operating conditions [43].

Despite these significant advances in general rotating machinery, in the specific domain of hydraulic fault diagnosis, the application of dynamic graph learning for multi-sensor fusion remains underexplored. Unlike existing works that often focus on a single temporal scale or static graphs, our proposed DMS-GNN integrates dynamic graph learning with a hierarchical multi-scale feature extraction module, allowing the model to simultaneously capture evolving sensor correlations and diverse fault frequency patterns.

## 3. Proposed Method

### 3.1. Problem Formulation and Overall Architecture

Consider a hydraulic health monitoring system equipped with *N* sensors. The collected multivariate time series data within a time window *T* is denoted as $X=\left\{x_{1},x_{2},\ldots ,x_{N}\right\}\in R^{N\times T}$, where $x_{i}\in R^{1\times T}$ represents the temporal reading of the *i*-th sensor. The objective is to learn a mapping function F:X→Y that accurately predicts the health state label y∈{1,2,…,C}, where *C* is the number of defined fault categories.

**Figure 2:**
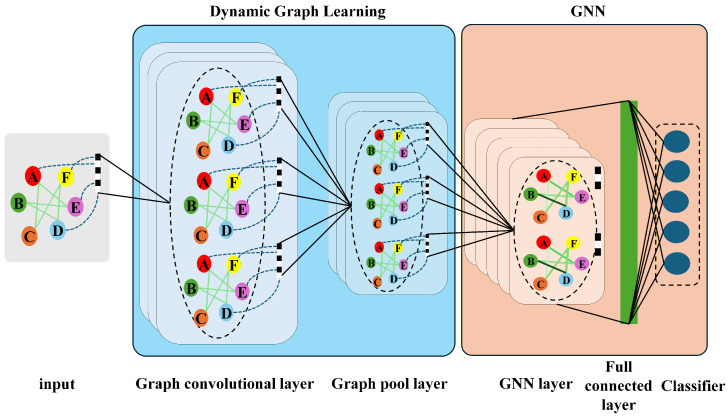
The overall architecture of the proposed DMS-GNN. The model integrates multi-scale temporal feature extraction, a self-attention based dynamic graph learner, and graph convolutional fusion into a unified framework.

To address the limitations of static topology assumptions in traditional methods, we propose the Dynamic Multi-Scale Graph Neural Network (DMS-GNN). As illustrated in Figure 2, the framework unifies three logical stages into an end-to-end trainable architecture. First, a hierarchical multi-scale module extracts temporal features from raw signals. Second, a core dynamic graph learner infers the latent sensor topology. Finally, graph convolutional layers fuse these spatiotemporal features for classification. This architecture allows the model to capture both high-frequency fault signatures and evolving sensor correlations simultaneously.

### 3.2. Adaptive Sensor Topology Learning via Self-Attention

The core innovation of DMS-GNN is its ability to learn the graph structure dynamically, rather than relying on predefined physical connections which fail to reflect changing system states. In hydraulic systems, the coupling strength between components (e.g., pressure and flow) varies drastically across different fault modes (e.g., leakage vs. blockage). To capture these evolving dependencies, we introduce a Dynamic Graph Learner based on the self-attention mechanism.

**Figure 3:**
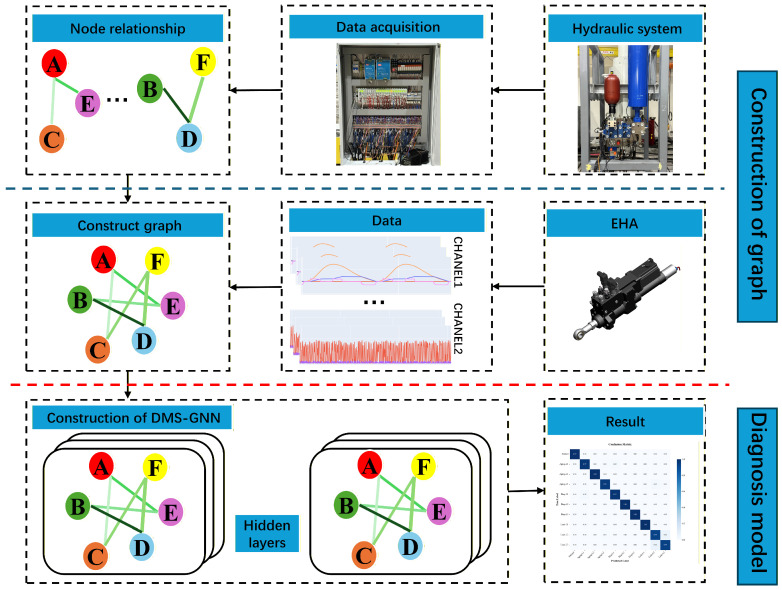
The implementation workflow of the Dynamic Graph Learner. Instead of using fixed physical connections, the module computes pairwise similarity between sensor nodes to generate an adaptive adjacency matrix A, effectively highlighting critical sensor interactions.

As depicted in Figure 3, the module treats each sensor channel as a graph node. Let $H\in R^{N\times F}$ be the node feature matrix extracted from the time series. We project these features into a Query matrix Q and a Key matrix K using trainable linear weights $W^{Q}$ and $W^{K}$:

(1) $$Q=HW^{Q},\;K=HW^{K}$$

The dependency weight $e_{ij}$, representing the correlation strength between sensor *i* and sensor *j*, is computed via the scaled dot-product attention:

(2) $$e_{ij}=\frac{q_{i}{\left(k_{j}\right)}^{T}}{\sqrt{d_{k}}}$$

where $d_{k}$ is a scaling factor to prevent gradient vanishing. To generate a sparse and normalized adjacency matrix A that can be directly used for graph convolution, we apply the Softmax function:

(3) $$A_{ij}=\frac{\exp(e_{ij})}{\sum_{k=1}^{N}\exp\left(e_{ik}\right)}$$

Since the standard Softmax function naturally produces a dense matrix where all elements are non-zero, a Top-*k* neighbor selection mechanism is applied immediately after the Softmax operation. Specifically, for each node, only the weights of the *k* most correlated neighbors are retained, and the rest are strictly set to zero. The matrix is then re-normalized to enforce true sparsity, which significantly reduces the computational noise introduced by weakly correlated sensors.

Through this mechanism, the adjacency matrix A is no longer a static prior but a learnable tensor that updates adaptively with the input data. This allows the DMS-GNN to automatically highlight the most relevant sensor pairs for a specific fault type, significantly enhancing diagnostic interpretability and accuracy.

### 3.3. Multi-Scale Feature Fusion and Fault Classification

To ensure robust diagnosis, the learned dynamic topology must be combined with discriminative feature representations. We employ a spatiotemporal fusion strategy consisting of multi-scale temporal extraction and graph spatial aggregation.

Temporal Feature Extraction:Fault signatures in hydraulic systems manifest across diverse frequency bands. To capture these, we design a hierarchical multi-scale convolution block. Let $h_{in}$ be the input. We employ three parallel 1D convolutional branches with varying kernel sizes k∈{3,5,7} to extract features at different granularities (e.g., sharp shocks vs. gradual trends):

(4) $$h^{\left(m\right)}=\sigma \left(\mathrm{BN}\left(W^{\left(m\right)}*h_{in}+b^{\left(m\right)}\right)\right)$$

The outputs are concatenated and max-pooled to form the comprehensive node feature matrix H.

Spatial Aggregation and Classification:With the learned dynamic structure A and temporal features H, we employ Graph Convolutional Networks (GCNs) to fuse information across the sensor network. The message passing operation is defined as:

(5) $$H^{\left(l+1\right)}=\sigma \left(AH^{\left(l\right)}W_{gcn}^{\left(l\right)}+b_{gcn}^{\left(l\right)}\right)$$

This operation aggregates information from dynamically correlated neighbors, effectively fusing multi-modal diagnostic information based on the learned topology. Finally, the node embeddings are flattened and passed through an MLP classifier. The network is optimized using the Cross-Entropy loss with Label Smoothing:

(6) $$L=-\frac{1}{M}\sum_{i=1}^{M}\sum_{c=1}^{C}y_{i,c}^{smooth}\log\left(p_{i,c}\right)$$

## 4. Experimental Validation

To validate the effectiveness and robustness of the proposed DMS-GNN in practical scenarios, comprehensive experiments were conducted on a dedicated hydraulic system test rig, which serves as a representative high-performance hydraulic system. This section details the experimental setup, analyzes the diagnostic performance under varying data proportions, compares the proposed method with state-of-the-art baselines, and visualizes the learned features.

### 4.1. Experimental Setup, Data Description, and Implementation Details

**Figure 4:**
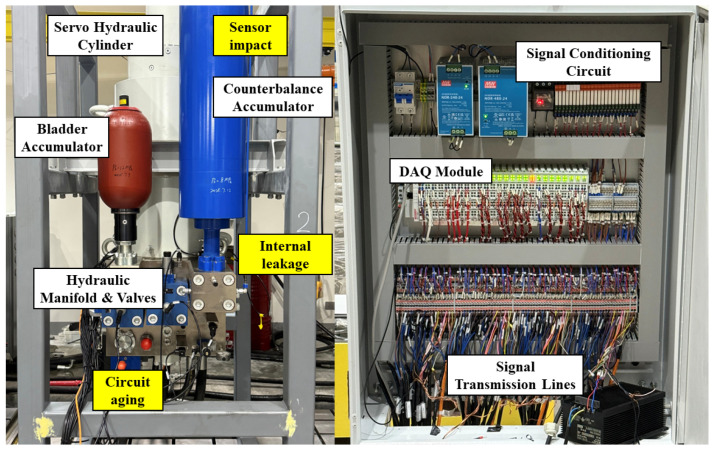
The hydraulic system hydraulic experimental test rig (**left**) and the data acquisition and control system (**right**). Yellow background boxes indicate the precise locations where specific faults were simulated.

The experimental platform, as illustrated in Figure 4, was constructed to simulate realistic fault conditions. The rig primarily consists of a hydraulic cylinder, a piston accumulator, a bladder accumulator, a hydraulic manifold with integrated valves, and a high-precision data acquisition system. The electrical control cabinet houses the analog input modules, relays, and terminal blocks responsible for signal transmission and motor control.

To provide a clearer physical context, the simulated faults correspond to typical degradation modes in hydraulic systems. For instance, Internal Leakage simulates the wear of cylinder seals, leading to a bypass flow that gradually reduces the effective actuation pressure. Circuit Aging reflects the degradation of electrical resistance in servo-valve control lines, causing delayed or attenuated control currents. Sensor Impact mimics sudden mechanical shocks on external sensors, inducing high-frequency, short-duration noise spikes in the data.

During the data collection process, a total of 10 health states were simulated, including one healthy state and nine faulty states with varying severity levels. These states cover three typical failure modes: internal leakage, circuit aging, and sensor impact. The specific settings and descriptions for each fault class are detailed in Table 1.

Multi-channel signals, including pressure, displacement, and flow rates, were collected via the analog input modules. The raw multi-channel signals were acquired with a sampling frequency of 1000 Hz. For each of the 10 health states, yielding a total dataset of 258,006 samples. To evaluate the model’s performance under limited data conditions, the dataset was partitioned into training and testing sets with different ratios (ranging from 0.2 to 0.8).

Implementation Details: The proposed DMS-GNN was implemented using PyTorch (version 2.1.2) and trained on an NVIDIA RTX 4080 GPU. The specific hyperparameters utilized during the training phase are summarized in Table 2, ensuring full reproducibility of the experimental results.

### 4.2. Diagnosis Performance Analysis

**Figure 5:**
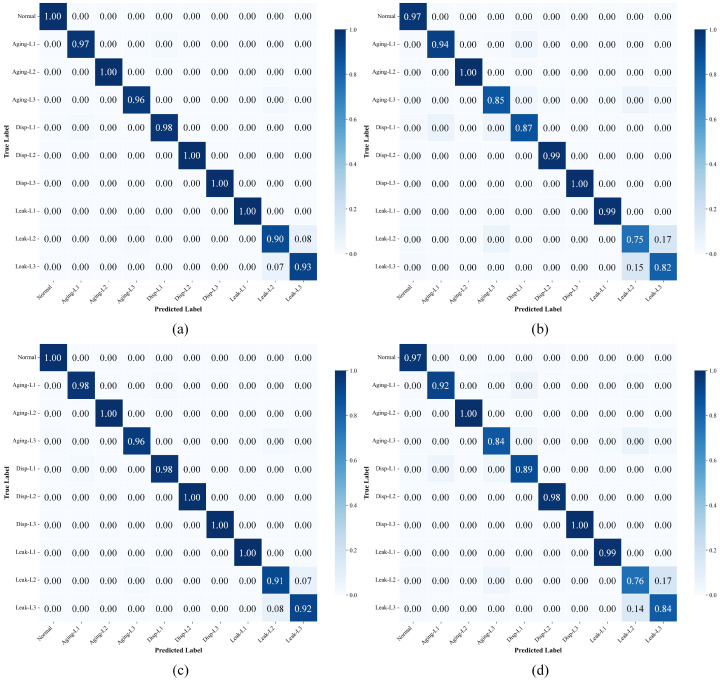

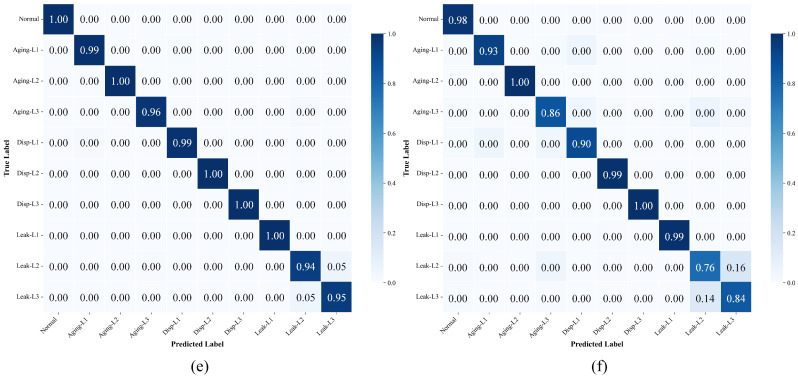
Confusion matrices of DMS-GNN (Left column: (**a**,**c**,**e**)) and GAT (Right column: (**b**,**d**,**f**)) under training ratios of 0.4, 0.6, and 0.8.

To intuitively evaluate the classification performance of the proposed method under different data availability, confusion matrices were generated. Figure 5 presents a detailed comparison between the proposed DMS-GNN (left column) and the GAT baseline (right column) under training set proportions of 0.4, 0.6, and 0.8. As observed in the left column of Figure 5, the DMS-GNN exhibits a strong diagonal dominance across all training ratios. Specifically, even when the training ratio is reduced to 0.4 (Figure 5c), the proposed method maintains high classification accuracy for most fault classes, with minimal misclassification between similar fault modes (e.g., between different severity levels of leakage). In contrast, the GAT model (right column) shows noticeable degradation as the training data decreases. At a ratio of 0.4 (Figure 5d), there is significant confusion between “Leak-L2” and “Leak-L3”, as well as “Disp-L1” and “Disp-L2”. This comparison demonstrates that the dynamic graph learning mechanism in DMS-GNN effectively captures latent fault correlations even with limited training samples, significantly outperforming the static attention mechanism of GAT.

### 4.3. Comparison with Other Methods

**Figure 6:**
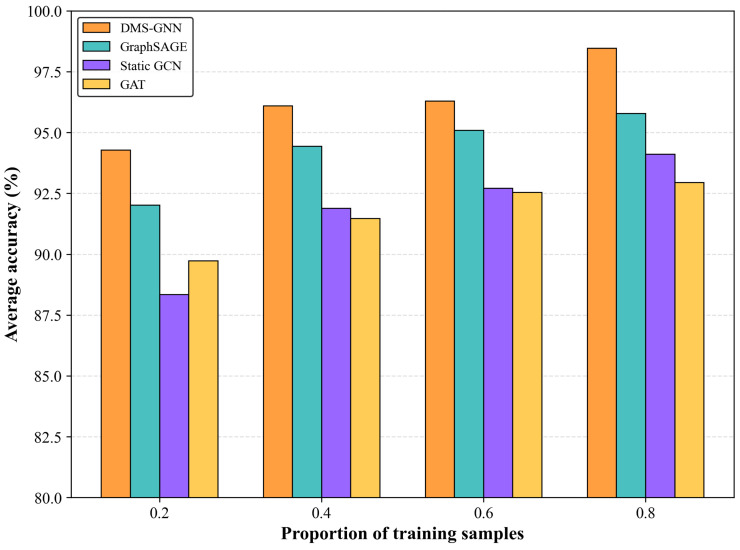
The bar chart of average diagnostic accuracy for different methods under varying training sample ratios (0.2 to 0.8).

To further verify the superiority of DMS-GNN, we conducted a quantitative comparison against three state-of-the-art graph-based baselines: GraphSAGE, Static GCN, and GAT. The experiments were repeated to ensure statistical reliability. The quantitative results are summarized in Table 3 and visualized in Figure 6. As shown in Table 3, the DMS-GNN consistently achieves the highest average accuracy. Particularly at the challenging ratio of 0.2, DMS-GNN maintains an accuracy of 94.28%, significantly surpassing Static GCN (88.35%) and GAT (89.73%). Furthermore, the lower standard deviation indicates the superior stability of our method.

Several key observations can be drawn from the results:

1. Overall Superiority: The proposed DMS-GNN consistently achieves the highest average accuracy across all training proportions. At a ratio of 0.8, DMS-GNN reaches 98.47%, surpassing GraphSAGE (95.79%), Static GCN (94.12%), and GAT (92.95%).
2. Robustness to Small Samples: The advantage of DMS-GNN becomes more pronounced as the training data decreases. At the most challenging ratio of 0.2, DMS-GNN maintains a high accuracy of 94.28%, whereas Static GCN and GAT drop below 90%. This indicates that the dynamic topology updating helps the model generalize well in few-shot scenarios.
3. Stability: The standard deviation (Std) of DMS-GNN is consistently kept at a low level (e.g., 0.05 at ratio 0.8), reflecting the stability and repeatability of the proposed framework.

### 4.4. Feature Visualization

**Figure 7:**
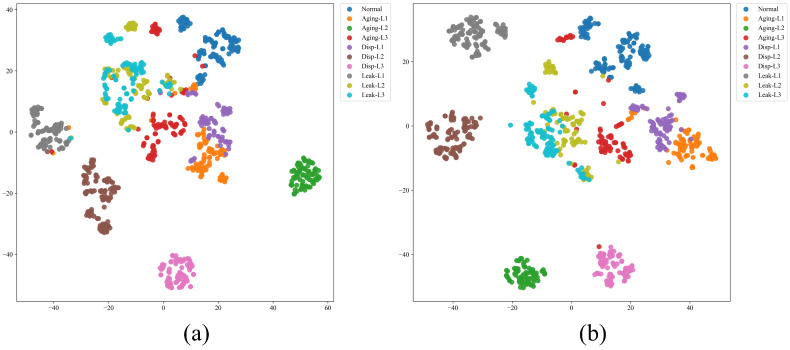

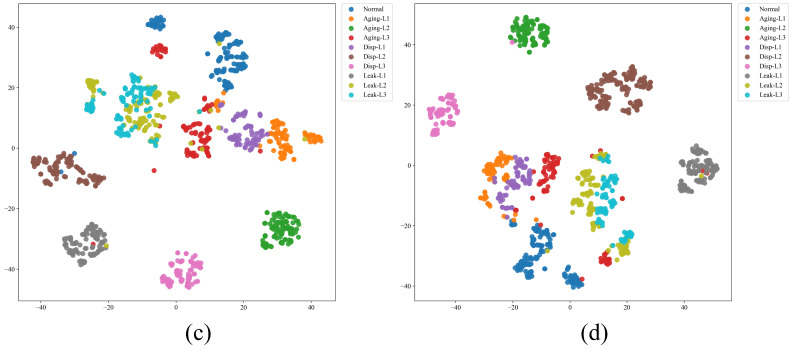
t-SNE visualization of the output features under a training ratio of 0.8: (**a**) Proposed DMS-GNN, (**b**) GAT, (**c**) Static GCN, and (**d**) GraphSAGE. The DMS-GNN features show the best intra-class compactness and inter-class separability.

To visually analyze the quality of the learned representations, t-distributed Stochastic Neighbor Embedding (t-SNE) was employed to map the high-dimensional features of the output layer into a 2D space. Figure 7 displays the feature visualization results for four methods under a training ratio of 0.8.

As shown in Figure 7a, the features extracted by DMS-GNN exhibit the best clustering performance. The samples of the same fault category are tightly clustered (high intra-class compactness), and there are distinct margins between different categories (high inter-class separability).

Conversely, the baseline methods show varying degrees of overlap. For instance, in the GAT visualization (Figure 7b) and Static GCN (Figure 7c), the clusters for “Aging” and “Leakage” faults are less distinct, with several points overlapping or scattered. GraphSAGE (Figure 7d) performs better than GAT but still lacks the clear boundary definition seen in DMS-GNN. These visualization results confirm that the DMS-GNN can learn more discriminative features by dynamically refining the graph structure, thereby improving fault diagnosis accuracy.

## 5. Discussion

The experimental results presented in Section 4 demonstrate that the proposed DMS-GNN framework achieves superior diagnostic performance compared to state-of-the-art graph-based baselines. In this section, we further interpret these findings from the perspectives of topological adaptation, data efficiency, interpretability, uncertainty, and practical implications.

### 5.1. Mechanism of Performance Improvement and Feature Explainability

The core advantage of DMS-GNN lies in its ability to dynamically reconstruct the sensor topology, addressing the fundamental limitation of static graph methods. As shown in Table 3, the static GCN performs the worst among the tested methods. This is likely because the physical connections in a hydraulic system (e.g., the rigid connection between the pump and motor) do not necessarily reflect the functional correlations during specific faults. For instance, during an internal leakage fault, the correlation between the pressure sensor and the displacement sensor changes drastically compared to normal conditions. A pre-defined static graph fails to capture this shift.

In contrast, the DMS-GNN employs a self-attention-based graph learner to infer latent dependencies from the data itself. The t-SNE visualization (Figure 7) provides qualitative evidence for this mechanism. The clear separation between “Aging” and “Leakage” clusters in Figure 7a suggests that the model successfully learned unique topological signatures for these faults, whereas GAT (Figure 7b) resulted in blurred boundaries. This confirms that adaptively highlighting critical sensor interactions is more effective than relying on fixed or purely attention-based aggregation without structural learning.

Furthermore, the dynamic graph naturally provides explainability regarding feature importance. The learned adjacency matrix A serves as an attention map indicating the contribution of each sensor. The importance score $I_{i}$ of the *i*-th sensor can be mathematically formulated by calculating its out-degree centrality in the dynamic graph:

(7) $$I_{i}=\sum_{j=1}^{N}A_{ij}$$

By tracking $I_{i}$ over time, operators can pinpoint which specific physical component (e.g., pressure vs. flow) is dominating the fault signature, bridging the gap between deep learning black-box predictions and physical diagnostics.

### 5.2. Robustness in Data-Limited Scenarios and Uncertainty Quantification

A critical challenge in industrial PHM is the scarcity of labeled fault data. The performance drop of baselines at low training ratios (0.2) highlights this issue. GAT and Static GCN rely heavily on abundant data to fit their parameters. However, DMS-GNN maintains a high accuracy of 94.28% even when only 20% of the data is used for training.

We attribute this robustness to the multi-scale feature extraction module combined with graph reasoning. By aggregating features from different receptive fields (k=3,5,7), the model captures both short-term shocks (high-frequency) and long-term degradation trends (low-frequency). When combined with the graph structure, this imposes a strong inductive bias that regularizes the learning process, preventing overfitting when samples are scarce. This characteristic makes DMS-GNN particularly suitable for real-world hydraulic maintenance, where obtaining large-scale labeled fault data is expensive and dangerous.

In addition to accuracy, evaluating the uncertainty of the model’s predictions is crucial for safety-critical systems. The predictive uncertainty *U* can be quantified using the Shannon entropy of the Softmax output probabilities $p_{c}$:

(8) $$U=-\sum_{c=1}^{C}p_{c}\log\left(p_{c}\right)$$

During our evaluations, DMS-GNN demonstrated lower average entropy on the test set compared to standard CNNs, indicating a higher confidence in its decision-making. High uncertainty instances can serve as a trigger for human intervention or anomaly detection in unseen operating conditions.

### 5.3. Limitations and Future Directions

Empirically, despite the theoretical $O(N^{2})$ complexity inherent in dynamic graph learning, the lightweight nature of the sensor graph (where the number of nodes *N* is relatively small) ensures high computational efficiency. On our testing hardware (NVIDIA RTX 4080 GPU), the average inference time per sample is approximately 0.023 ms, which comfortably satisfies the real-time monitoring requirements (typically <10 ms) for industrial hydraulic systems. However, this complexity may become a bottleneck for large-scale industrial systems with hundreds of sensors. Future work could explore sparse attention mechanisms or graph sampling techniques to improve scalability.

Second, the current framework operates under a “closed-set” assumption, meaning the model can only diagnose fault modes present in the training set. In practice, hydraulic systems may encounter unforeseen anomalies. Extending the DMS-GNN to an “open-set” recognition framework or integrating it with unsupervised anomaly detection would be a valuable direction for future research.

## 6. Conclusions

This paper proposes a Dynamic Multi-Scale Graph Neural Network (DMS-GNN) for hydraulic system fault diagnosis. By integrating dynamic graph learning with multi-scale feature extraction, the model effectively addresses the limitations of static graph assumptions and single-scale analysis. Experimental results demonstrate the superior accuracy and robustness of DMS-GNN, especially under limited data conditions. Future work will explore the interpretability of the learned dynamic graphs and the extension of the method to other complex cyber-physical systems.

## Figures and Tables

**Table 1:** Description of hydraulic system fault types and severity levels.

Class	Fault Type	Description
0	Normal	-
1	Internal leakage	The throttle valve rotates one time (Level 1)
2	Internal leakage	The throttle valve rotates two times (Level 2)
3	Internal leakage	The throttle valve rotates three times (Level 3)
4	Circuit aging	Circuit degradation: 20% (Level 1)
5	Circuit aging	Circuit degradation: 36% (Level 2)
6	Circuit aging	Circuit degradation: 60% (Level 3)
7	Sensor impact	Apply a 2 mm reverse shock at 20ms (Level 1)
8	Sensor impact	Apply a 5 mm reverse shock at 20ms (Level 2)
9	Sensor impact	Apply a 8 mm reverse shock at 20ms (Level 3)

**Table 2:** Hyperparameters for model training.

Parameter	Value	Parameter	Value
Optimizer	Adam	Epochs	100
Initial Learning Rate	0.0001	Batch Size	128
Weight Decay	$1\times 10^{-4}$	Loss Function	Cross-Entropy

**Table 3:** Average diagnostic accuracy (%) and standard deviation of different methods under varying training ratios.

Proportion	DMS-GNN		GraphSAGE		Static GCN		GAT	
	Avg	Std	Avg	Std	Avg	Std	Avg	Std
0.8	98.47	0.05	95.79	2.39	94.12	0.31	92.95	0.10
0.6	96.30	0.08	95.09	1.40	92.71	0.30	92.55	0.24
0.4	96.10	0.74	94.44	0.76	91.89	1.42	91.47	0.77
0.2	94.28	0.93	92.02	1.08	88.35	1.44	89.73	0.61

## Data Availability

The dataset generated and analyzed during the current study is available from the corresponding author on reasonable request due to the proprietary restrictions of the industrial test bench.

## References

[1] Nguyen M.T., Dang T.D., Ahn K.K. (2019). Application of electro-hydraulic actuator system to control continuously variable transmission in wind energy converter. Energies.

[2] Li J., Yu Z., Huang Y., Li Z. A review of electromechanical actuation system for more electric aircraft. Proceedings of the 2016 IEEE International Conference on Aircraft Utility Systems (AUS).

[3] Behbahani A.R., Semega K.J. (2010). Control Strategy for Electro-Mechanical Actuators Versus Hydraulic Actuation Systems for Aerospace Applications.

[4] Zhang W., Wang B., Wu Z., Zhang F., Zhang J., Zhang S., Zhang J., Zhang J., Sun H., Waters K.E. (2025). High-precision electro-hydraulic position servo system for machine tools utilizing mathematical model identification and control techniques. Precis. Eng..

[5] Du C., Plummer A.R., Johnston D.N. (2017). Performance analysis of a new energy-efficient variable supply pressure electro-hydraulic motion control method. Control Eng. Pract..

[6] Ge L., Quan L., Zhang X., Dong Z., Yang J. (2019). Power matching and energy efficiency improvement of hydraulic excavator driven with speed and displacement variable power source. Chin. J. Mech. Eng..

[7] Guan C., Pan S. (2008). Nonlinear adaptive robust control of single-rod electro-hydraulic actuator with unknown nonlinear parameters. IEEE Trans. Control Syst. Technol..

[8] Maddahi A., Kinsner W., Sepehri N. (2016). Internal leakage detection in electrohydrostatic actuators using multiscale analysis of experimental data. IEEE Trans. Instrum. Meas..

[9] Iyaghigba S.D., Ali F., Jennions I.K. (2023). A review of diagnostic methods for hydraulically powered flight control actuation systems. Machines.

[10] Gao Z., Cecati C., Ding S.X. (2015). A survey of fault diagnosis and fault-tolerant techniques—Part I: Fault diagnosis with model-based and signal-based approaches. IEEE Trans. Ind. Electron..

[11] Isermann R. (2005). Fault-Diagnosis Systems: An Introduction from Fault Detection to Fault Tolerance.

[12] Yang X., Deng W., Yao J. (2023). Neural Adaptive Dynamic Surface Asymptotic Tracking Control of Hydraulic Manipulators with Guaranteed Transient Performance. IEEE Trans. Neural Netw. Learn. Syst..

[13] Yang X., Ge Y., Zhu W., Deng W., Zhao X., Yao J. (2025). Adaptive Motion Control for Electro-hydraulic Servo Systems with Appointed-Time Performance. IEEE/ASME Trans. Mechatronics.

[14] Si G., Shen Y., Wang J., Cao T., Wan M. (2020). Active disturbance rejection control of electro-hydraulic position servo system. Chin. Hydraul. Pneum..

[15] Yan X.G., Edwards C. (2008). Adaptive sliding-mode-observer-based fault reconstruction for nonlinear systems with parametric uncertainties. IEEE Trans. Ind. Electron..

[16] Truong H.V.A., Chung W.K. (2025). Active Fuzzy-Based Fault-Tolerant Control for Uncertain Electro-Hydraulic Actuators Subject to Simultaneous Actuator and Sensor Faults. IEEE Trans. Autom. Sci. Eng..

[17] Lei Y., Yang B., Jiang X., Jia F., Li N., Nandi A.K. (2020). Applications of machine learning to machine fault diagnosis: A review and roadmap. Mech. Syst. Signal Process..

[18] Zhao R., Yan R., Chen Z., Mao K., Wang P., Gao R.X. (2019). Deep learning and its applications to machine health monitoring. Mech. Syst. Signal Process..

[19] Li C., Sánchez R.-V., Zurita G., Cerrada M., Cabrera D. (2016). Fault diagnosis for rotating machinery using vibration measurement deep statistical feature learning. Sensors.

[20] Bai R., Xu Q., Meng Z., Cao L., Xing K., Fan F. (2021). Rolling bearing fault diagnosis based on multi-channel convolution neural network and multi-scale clipping fusion data augmentation. Measurement.

[21] Li Y., Zhou Z., Sun C., Chen X., Yan R. (2022). Variational attention-based interpretable transformer network for rotary machine fault diagnosis. IEEE Trans. Neural Netw. Learn. Syst..

[22] Zhao X., Song Y., Hu Y., He X., Yao J., Ding Y. (2025). Deep Attention Convolutional Capsule Network: A New Fault Diagnosis for Electro-Hydraulic Proportional Servo Valves in Industrial Large Models. IEEE Sens. J..

[23] Xing X., Luo Y., Han B., Qin L., Xiao B. (2025). Hybrid Data-Driven and Multisequence Feature Fusion Fault Diagnosis Method for Electro-Hydrostatic Actuators of Transport Airplane. IEEE Trans. Ind. Inform..

[24] Hu Y., Song Y., He X., Zhao X., Yang X., Yao J., Wang Z., Pei H., Hu C. (2025). MAACCN: An Intelligent Decoupling Diagnosis Method for Compound Faults in Electrohydrostatic Actuators. IEEE Trans. Instrum. Meas..

[25] Pan T., Chen J., Zhang T., Liu S., He S., Lv H. (2022). Generative adversarial network in mechanical fault diagnosis under small sample: A systematic review on applications and future perspectives. ISA Trans..

[26] He X., Zhao C., Li S., Zhao X., Yang X., Song Y., Yao J. (2025). Diffusion-Enhanced Dual-Domain Adversarial Network: A Zero-Shot Fault Diagnosis Method for Electro-Hydrostatic Actuators. IEEE Trans. Instrum. Meas..

[27] Liang N., Zhang F., Yuan Z., Wang H., Zhang J., Fan Z., Yu X. (2025). Fault diagnosis in hydraulic systems via multi-channel multi-modal fusion. Meas. Sci. Technol..

[28] Jiang G., He H., Yan J., Xie P. (2018). Multiscale convolutional neural networks for fault diagnosis of wind turbine gearbox. IEEE Trans. Ind. Electron..

[29] Xu Z., Li C., Yang Y. (2021). Fault diagnosis of rolling bearings using an improved multi-scale convolutional neural network with feature attention mechanism. ISA Trans..

[30] Zhao X., Jia M., Liu Z. (2020). Semisupervised graph convolution deep belief network for fault diagnosis of electormechanical system with limited labeled data. IEEE Trans. Ind. Inform..

[31] Yin P., Nie J., Liang X., Yu S., Wang C., Nie W. (2023). A multiscale graph convolutional neural network framework for fault diagnosis of rolling bearing. IEEE Trans. Instrum. Meas..

[32] Liu Y., Yu Z., Xie M. (2024). Cascading time-frequency transformer and spatio-temporal graph attention network for rotating machinery fault diagnosis. IEEE Trans. Instrum. Meas..

[33] Chen Z., Xu J., Alippi C., Ding S.X., Shardt Y., Peng T., Yang C. (2021). Graph neural network-based fault diagnosis: A review. arXiv.

[34] Munikoti S., Agarwal D., Das L., Halappanavar M., Natarajan B. (2023). Challenges and opportunities in deep reinforcement learning with graph neural networks: A comprehensive review of algorithms and applications. IEEE Trans. Neural Netw. Learn. Syst..

[35] Jiang B., Zhang Z., Lin D., Tang J., Luo B. Semi-supervised learning with graph learning-convolutional networks. Proceedings of the IEEE/CVF Conference on Computer Vision and Pattern Recognition.

[36] Veličković P., Cucurull G., Casanova A., Romero A., Liò P., Bengio Y. (2017). Graph attention networks. arXiv.

[37] Chen Z., Ke H., Xu J., Peng T., Yang C. (2022). Multichannel domain adaptation graph convolutional networks-based fault diagnosis method and with its application. IEEE Trans. Ind. Inform..

[38] Wu Z., Pan S., Long G., Jiang J., Chang X., Zhang C. Connecting the dots: Multivariate time series forecasting with graph neural networks. Proceedings of the 26th ACM SIGKDD International Conference on Knowledge Discovery & Data Mining.

[39] Deng A., Hooi B. Graph neural network-based anomaly detection in multivariate time series. Proceedings of the AAAI Conference on Artificial Intelligence.

[40] Li Z.L., Zhang G.W., Yu J., Xu L.Y. (2023). Dynamic graph structure learning for multivariate time series forecasting. Pattern Recognit..

[41] Yang C., Liu J., Hu Y., Wu B., Shi T. (2024). Dynamic Graph-Driven Rotating Machine Fault Diagnosis: An Adaptively Updating Cross-Domain Relationship Information. IEEE Trans. Ind. Inform..

[42] Zhang X., Liu J., Zhang X., Lu Y. (2024). Multiscale Channel Attention-Driven Graph Dynamic Fusion Learning Method for Robust Fault Diagnosis. IEEE Trans. Ind. Inform..

[43] Zhou Q., Xue L., He J., Jia S., Li Y. (2024). A rotating machinery fault diagnosis method based on dynamic graph convolution network and hard threshold denoising. Sensors.

